# Online Survival Analysis Software to Assess the Prognostic Value of Biomarkers Using Transcriptomic Data in Non-Small-Cell Lung Cancer

**DOI:** 10.1371/journal.pone.0082241

**Published:** 2013-12-18

**Authors:** Balázs Győrffy, Pawel Surowiak, Jan Budczies, András Lánczky

**Affiliations:** 1 Research Laboratory of Pediatrics and Nephrology, Hungarian Academy of Sciences, Budapest, Hungary; 2 Department of Histology and Embryology, Wroclaw Medical University, Wrocław, Poland; 3 Institut für Pathologie, Charité – Universitätsmedizin Berlin, Berlin, Germany; H. Lee Moffitt Cancer Center & Research Institute, United States of America

## Abstract

In the last decade, optimized treatment for non-small cell lung cancer had lead to improved prognosis, but the overall survival is still very short. To further understand the molecular basis of the disease we have to identify biomarkers related to survival. Here we present the development of an online tool suitable for the real-time meta-analysis of published lung cancer microarray datasets to identify biomarkers related to survival. We searched the caBIG, GEO and TCGA repositories to identify samples with published gene expression data and survival information. Univariate and multivariate Cox regression analysis, Kaplan-Meier survival plot with hazard ratio and logrank P value are calculated and plotted in R. The complete analysis tool can be accessed online at: www.kmplot.com/lung. All together 1,715 samples of ten independent datasets were integrated into the system. As a demonstration, we used the tool to validate 21 previously published survival associated biomarkers. Of these, survival was best predicted by CDK1 (p<1E-16), CD24 (p<1E-16) and CADM1 (p = 7E-12) in adenocarcinomas and by CCNE1 (p = 2.3E-09) and VEGF (p = 3.3E-10) in all NSCLC patients. Additional genes significantly correlated to survival include RAD51, CDKN2A, OPN, EZH2, ANXA3, ADAM28 and ERCC1. In summary, we established an integrated database and an online tool capable of uni- and multivariate analysis for *in silico* validation of new biomarker candidates in non-small cell lung cancer.

## Introduction

Although lung cancer treatment options have improved significantly in the last decade leading to better survival for patients with every stage of the disease, it is still leading cancer related deaths in the United States with 160 thousand deaths each year [1]. With approximately 85% of all cases the most common type of lung cancer is non-small cell lung cancer (NSCLC), which includes adenocarcinoma, squamous cell carcinoma, large cell carcinoma, and bronchioloalveolar carcinoma [2]. Similarly to other cancer entities we can expect new molecular subtypes to emerge in the future, as it is now well accepted that the light microscopy based histologic subdivision uses only one of many phenotypic manifestations of the genetic changes that underlie lung cancer development [2].

The identification of genes whose altered expression is associated with survival differences might enclose the knowledge to pinpoint those which could serve as indicators of the tumor's biological state. In essence there are two possible scenarios for this: such biomarker can either be an individual gene or a signature comprising a set of genes. While numerous individual genes associated with survival have been published in the last thirty years, new microarray-based multigene molecular prognostic models using genomic signatures have only emerged in the last ten years [3], [4], [5], [6], [7], [8], [9], [10], [11], [12], [13], [14], [15], [16], [17], [18], [19]. A pre-requisite for the reproducibility of such genomic signatures is the availability of raw data, which was only ensured by publications of the last six years [9], [10], [11], [12], [13], [14], [15], [16], [17], [18]. Remarkably, in two cases not the signature as a whole, but genes as each individually important prognostic markers have been identified [15], [19].

The initial discovery of a prognostic marker must be followed by several validation studies. Then, the results of these are usually synthesized in a meta-analysis including a large number of preferably more than thousand patients. Here, by uniting relevant data from several studies, statistical power is increased and more accurate estimates can be achieved. Several previous meta-analyses endeavored to perform such a meta-analysis of previous studies for solitary gene candidates including VEGF [20], MMP9 [21], cyclin E [22], survivin [23] and CDK1 [24].

Here, we integrated available genome-level transcriptomic datasets and then used this database to perform a meta-analysis of previously suggested survival associated biomarker-candidates. We also set up a global portal for such meta-analysis enabling express validation of new candidates without large-scale bioinformatic effort in an automated framework.

## Materials and Methods

### Construction of lung cancer microarray database

We explored the Cancer Biomedical Informatics Grid (caBIG, http://cabig.cancer.gov/, microarray samples are published in the caArray project), the Gene Expression Omnibus (GEO, http://www.ncbi.nlm.nih.gov/geo/) and The Cancer Genome Atlas (TCGA, http://cancergenome.nih.gov) to identify lung cancer datasets using the keywords “lung”, “cancer”, “small-cell”, “NSCLC”, “survival”, “GPL96”, “GPL3921” and “GPL570” (and the alternative names of the microarray platforms). The search was restricted to publications with simultaneously available microarray gene expression data and published clinical characteristics including survival. To test randomness, a pairwise rank test was performed for the collected clinical data including age, sex, smoking history, histology, stage, grade, success of surgery, radiotherapy and applied chemotherapy for all patients in WinStat 2013. For the pairwise rank test, the samples were first sorted according to datasets. Then, each sample (“X”) in the series was compared with all values which occur later in the list of all samples (“Y”) - assuming randomness, the probability of X>Y is 1/2. The correlations between clinical variables and survival were investigated and Kaplan-Meier plots for these were plotted using WinStat 2013. Among the different microarray platforms, Affymetrix HG-U133A (GPL96), HG-U133 Plus 2.0 (GPL570) and HG-U133A 2.0 (GPL3921) were included, because these are regularly used and because these arrays have 22,277 probe sets in common. The use of the same probe sets enables to measure the same gene with similar accuracy, relative scale and dynamic range.

To avoid potential bias due to array errors, we have performed a quality check for all arrays. In this, the background (between 19 and 218), the raw Q (between 0.5 and 14), the percentage of present calls (over 30%), the presence of bioB-/C-/D- spikes, the GAPDH 3′to 5′ ratio (below 4.3) and the beta-actin 3′ to 5′ ratio (below 18) were checked. The threshold values correspond to the 95% range of the arrays as described previously [25]. Quality control was not possible for GSE4573 as for this dataset only the MAS5 normalized data was available. A filtering was added to the database to exclude potentially biased arrays. Additionally, we compared all microarray files using the ranked expression of all genes to spot microarrays re-published in different studies.

### Set-up of server for online survival calculation

The unprocessed.CEL files were MAS5 normalized in the R environment (http://www.r-project.org) using the simpleaffy library (http://bioinformatics.picr.man.ac.uk/simpleaffy/). We have selected MAS5 for normalization as it ranked among the best normalization methods when contrasted to the results of RT-PCR measurements in our previous study [26]. Moreover, MAS5 can be applied to single arrays, enabling seamless future extensions of the database. For the complete database, only the common probes measured in all three array platforms were retained (n = 22,277). Then, a second scaling normalization was performed to center the mean expression for each array to 1000 - this technique can significantly reduce batch effects. Gene expression and clinical data were integrated using PostgreSQL, an open source object-relational database system (http://www.postgresql.org/).

To assess the prognostic value of a gene, each percentile (of expression) between the lower and upper quartiles were computed and the best performing threshold was used as the final cutoff in a univariate Cox regression analysis. Histology, grade, stage, gender and smoking history can be used in the multivariate analysis. However, the multivariate analysis uses less patients as the univariate analysis because not each patients has all clinical information. Kaplan-Meier survival plot and the hazard ratio with 95% confidence intervals and logrank P value were calculated and plotted in R using the “survplot” function of the “survival” Bioconductor package. The R script used by the software to perform the Kaplan-Meier analysis and to identify the best cutoff is available as **R script S1**.

The entire computational pathway is made accessible for re-analysis in a platform independent online available software running on a Debian Linux (http://www.debian.org) server powered by Apache (http://www.apache.org). The scripts on the server-side were developed in PHP, these control the user interface, the requests and the delivery of the results. The RODBC package provides a middleware layer between R and the PostgreSQL database. This platform can be reached over the internet via http://www.kmplot.com/lung.

### Validation of previously published survival associated biomarkers

A Pubmed search was performed to identify lung cancer survival associated biomarkers using all combinations of the keywords “lung cancer”, “NSCLC”, “adenocarcinoma”, “squamous cell carcinoma”, “survival”, “gene expression”, “signature” and “meta analysis”. Only studies published in English were included. Eligibility criteria also included the investigation of the biomarker in at least 50 patients - biomarkers described in experimental models only were omitted. For each gene/signature the exact conditions in which it was identified have been retrieved, and these have been used as filtering when selecting the patients for the survival analysis.

To visualize the performance of the various biomarkers in datasets including different number of patients, we have generated funnel plots depicting the hazard ratio (and confidence intervals) on the horizontal axis vs. the sample size on the vertical axis for each dataset. We also added an option to the online interface to simultaneously perform the analysis in each of the individual datasets. Finally, significance was set at p<0.01.

## Results

### Construction of combined lung cancer microarray database

**Figure 1:**
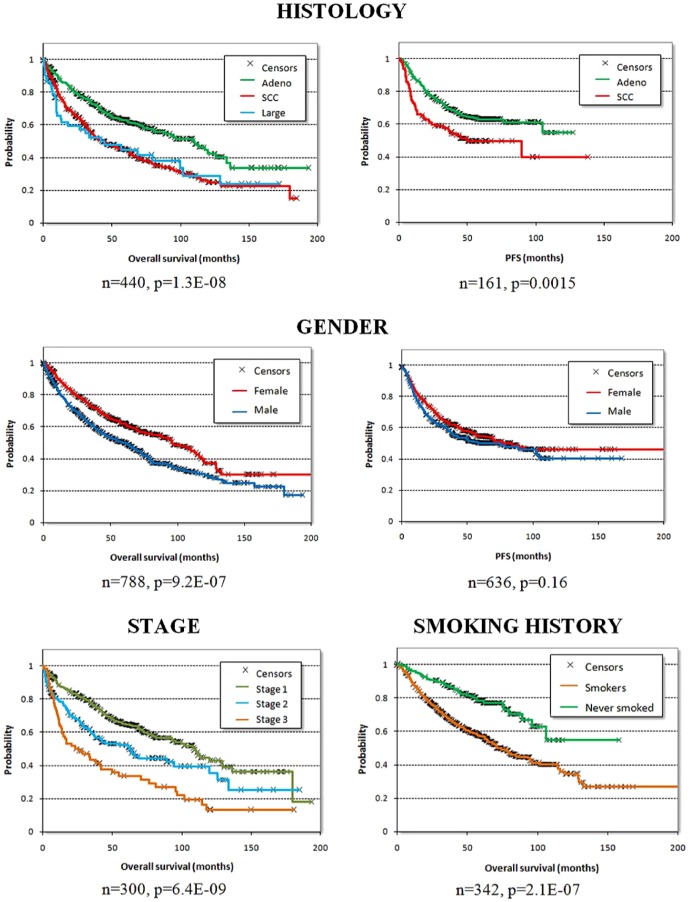
Survival characteristics of the patients included in the database including histology of adenocarcinoma (adeno), squamous cell carcinoma (SCC) and large cell carcinoma (large), gender, stage (only with overall survival) and smoking history.

We identified all together 1,715 patients, 1,120 in seven GEO datasets, 133 patients in TCGA and 462 patients in caArray. There were no samples repeatedly published. One sample (GSM370984) failed two parameters in the quality control - this array was excluded from all analyses. Additionally, in 215 arrays one parameter was out of the 95% range of all arrays - these arrays can be excluded from analyses by selecting the “exclude outlier arrays” in the online interface. Overall survival was published for 1,405 patients and time to first progression was published for 764 patients. We have collected age, sex, smoking history, histology, stage, grade, success of surgery, radiotherapy and applied chemotherapy for all patients - none of these parameters was significant in the pairwise rank test indicating random distribution of the data. A summary of these clinical properties for each dataset used is presented in **Table 1**. The survival of the patients stratified by subtype, gender, smoking history and stage is presented in **Figure 1**.

**Table 1:** Clinical characteristics of the datasets included in the analysis.

Dataset	Platform	Reference	Sample size	Median follow-up (OS)	No. of deaths	Median follow-up (FP)	No. of progressions	Age	Sex (% male)	% of never smokers	Histology (% A/S/L)	Stage (% 1/2/3/4)	% surgical margins negative	Grade (% poor/moderate/well)	% chemotherapy	% radiotherapy
GSE4573	GPL96	[9]	130	34.5	67	-	-	67±9.8	63%	3.1%	0/100/0	56/26/18/0	-	17/71/12	-	-
GSE14814	GPL96	[10]	90	5.4	38	-	-	62±8.5	74%	-	31/58/11	50/50/0/0	-	-	56%	-
GSE8894	GPL570	[16]	138	-	-	36	69	62±10	75%	-	46/54/0	-	-	-	-	-
GSE19188	GPL570	[11]	156	30.4	50	-	-	-	75%	-	49/30/21	-	-	-	-	-
GSE3141	GPL570	[12]	109	31.1	58	-	-	-	-	-	52/48/0	-	-	-	-	-
GSE31210	GPL570	[13]	246	58.2	35	54.4	64	60±8.1	47%	50%	100/0/0	74/26/0/0	90%	-	-	-
caArray	GPL96	[17]	462	45.8	257	28	219	64±10	51%	14%	-	-	98%	39/47/14	27%	21%
TCGA	GPL3921	[18]	133	18.3	30	-	-	66±9.3	67%	7.5%	0/100/0	-	95%	-	-	-
GSE29013	GPL570	[14]	55	32.9	18	31.4	28	64±8.7	69%	3.6%	55/45/0	44/25/31/0	-	-	62%	-
GSE37745	GPL570	[15]	196	42.5	145	-	-	64±9.2	55%	-	54/37/12	66/18/14/2	-	-	-	-
Entire database:			1715	40	698/1443	37	380/821	64±10	58% (n = 886)	17.8% (n = 187)	50/45/5	63/27/10/1	95% (n = 705)	34/53/13	29% (n = 178)	21% (n = 73)

OS: overall survival, FP: first progression, A/S/L: adenocarcinoma/squamous cell carcinoma/large cell carcinoma.

### Set-up of online survival analysis platform

We have employed Kaplan-Meier plots to visualize the association between the gene under investigation and survival. Before analysis, the patients were filtered using the available clinical parameters to include only those patients where the relevance of the gene is to be assessed. Besides filtering options specific for clinical parameters, we implemented an algorithm which includes the use of all percentiles between the lower and upper quartile to identify the best performing cutoff.

To our knowledge, present development is the very first system enabling real-time multivariate survival analysis of genes in available transcriptomic cohorts.

### Validation of previously published NSCLC biomarkers

**Figure 2:**
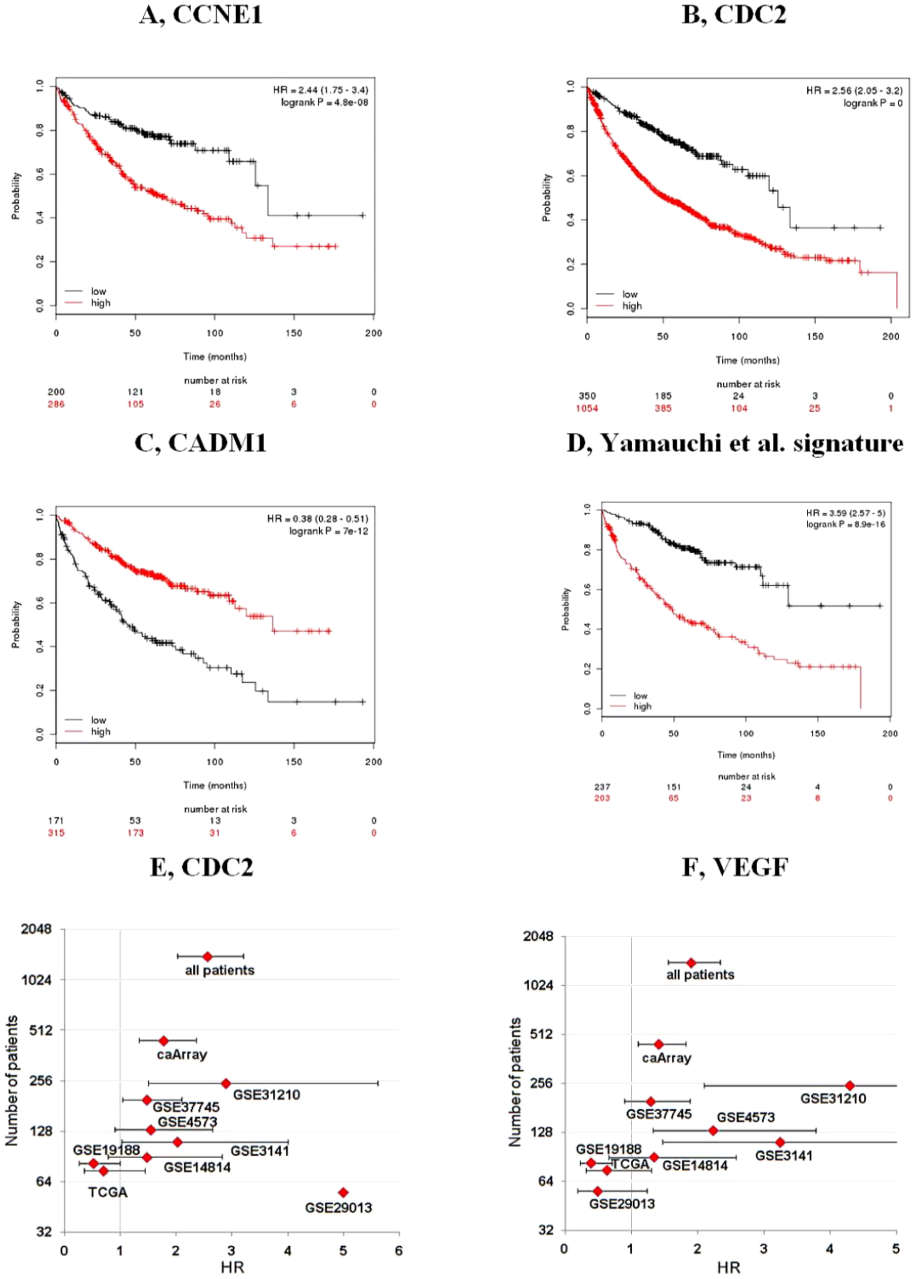
Validation of 29 previously published NSCLC biomarkers. Meta-analysis of these genes and signatures in the respective sample cohort yielded CCNE1, CDC2 and CADM1 as the best performing individual genes **(A–C)** and the signature of Yamauchi et al. **(D)**. A funnel plot depicting the hazard ratios (with confidence intervals) versus sample number for CDC2 and VEGF shows more reliable estimation with larger database sizes **(E–F)**.

We identified 21 previously published survival associated individual genes and 7 gene expression signatures (listed in **Table S1**). Each of these biomarker candidates were investigated in a cohort having similar clinical characteristics as the patients in which they were originally described. For genes measured by several probe sets on the microarrays, those with the highest quality were used (high quality: average expression over 500 or maximal expression over 1000, low quality: average expression below 100, intermediate: all other probes). In case there were several high quality probes then the best performing was used. The analysis results are presented in **Table 2** and **Figure 2**.

**Table 2:** Performance of previously published biomarker candidates associated with survival in non-small-cell lung cancer.

Gene	Literature data				Meta-analysis results					
Symbol	Ref.	n	Method used	Cohort	Probe ID*	n ✠	Cutoff ✠	HR✠	p value: univariate	p value: multivariate
**Genes examined in a meta-analysis**										
VEGF	[20]	5386	IHC, RT-PCR	NSCLC	211527_x_at	1404	244	1.9	3.3e-10	<1e-16
MMP9	[21]	2029	IHC, RT-PCR	NSCLC	203936_s_at	1404	1865	1.21	0.012	-
				ADE		486	734	1.51	0.02	-
CCNE1 *cyclin E*	[22]	2606	IHC	NSCLC	213523_at	1404	276	1.59	2.3e-09	0.0096
				ADE		486	167	2.44	4.8e-08^#^	0.0013
BIRC5 *survivin*	[23]	2703	IHC, FISH RT-PCR	NSCLC stage 2	202095_s_at	185	295	1.56	0.077	-
CDC2 *CDK1*	[24]	2731	IHC, RT-PCR	NSCLC	210559_s_at	1404	266	2.56	<1e-16^#^	0.0019
**Genes identified in original studies**										
CADM1	[15]	617	Array + IHC	ADE	209031_at	486	1793	0.38	7e-12^#^	0.0001
CEA	[33]	97	IHC	NSCLC	206199_at	1404	110	1.21	0.02	-
RAD51	[34]	383	IHC	NSCLC	205023_at	1404	44	1.4	2.4e-05	0.24
				ADE		486	34	1.36	0.046	-
				SCC		421	45	1.2	0.18	-
CDKN2A *P16*	[35]	106	IHC	NSCLC	209644_x_at	1404	1382	1.65	1.8e-09	0.12
				ADE		486	486	2.23	6.8e-08	0.012
OPN	[36]	25	IHC	All patients	209875_s_at	1404	4151	1.5	2.8e-06	0.0001
	[37]	82	RT-PCR	NSCLC surgical margin neg.		704	4101	1.93	1.5e-06	0.0032
EZH2	[38]	106	IHC	NSCLC stage 1	203358_s_at	440	600	2.07	2.6e-06	0.32
IFNAR2	[39]	113	IHC	NSCLC PFS	204785_x_at	764	799	1.41	0.0012	0.05
ANXA3	[40]	125	MS, 2D-DIGE	ADE	209369_at	486	811	0.49	9.2e-07	0.0093
S100A4	[41]	400	IHC	SCC	203186_s_at	421	2844	1.24	0.12	-
ADAM28	[42]	90	ELISA	NSCLC	205997_at	1404	143	0.69	8.3e-06	0.003
XIAP	[43]	144	IHC	NSCLC	206536_s_at	1404	85	0.86	0.071	-
XAF1	[44]	51	RT-PCR	SCC	206133_at	421	253	0.72	0.025	-
CD24	[45]	267	IHC	ADE	209772_s_at	486	618	2.45	3.6e-10	<1e–16
ERCC1	[46]	51	RT-PCR	NSCLC	203719_at	1404	685	1.65	1.4e-10	<1e-16
HER2	[47]	83	RT-PCR	NSCLC	216836_s_at	1404	898	1.25	0.0057	0.12
CD82	[48]	151	RT-PCR	NSCLC	203904_x_at	1404	506	1.27	0.0029	0.09
**Gene expression signatures identified using microarrays**										
139-gene	[13]	253	Array	NSCLC stage I	see Table S1	440	3368.7	3.59	8.9e-16^#^	<1e-16
59-gene	[14]	100	Array	NSCLC	see Table S1	1404	4038.6	0.66	9.9e-08	0.035
15-gene	[10]	133	Array + RT-PCR	NSCLC + chemo	see Table S1	173	573.7	0.6	0.042	-
50-gene	[9]	129	Array + RT-PCR + IHC	SCC	see Table S1	421	754.3	0.65	0.0016	0.0023
17-gene	[11]	91	Array	NSCLC	see Table S1	1404	618.3	1.27	0.0027	0.48
6-gene	[16]	138	Array + RT-PCR	NSCLC PFS	see Table S1	764	543.5	0.77	0.017	-
38-gene	[17]	462	Array	ADE	see Table S1	468	437.7	0.64	0.0031	0.092

ADE: adenocarcinoma; SCC: squamous cell carcinoma; 2D-DIGE: two-dimensional difference gel electrophoresis; MS: mass spectrometry; n: number of tumor samples included in the study; *highest quality probe, when several high quality probes then the best performing; ^#^ see Figure 2. for the survival plots; ✠ of the univariate analysis; multivariate: using those two parameters where most data was available (histology and gender for NSCLC, gender and stage for adenocarcinoma and squamous cell carcinoma). Multivariate analysis was performed only for biomarker candidates significant at p<0.01 in the univariate analysis.

## Discussion

The importance of cancer biomarkers is highlighted by the success of the HER2 gene in breast cancer. High HER2 expression was first a marker of worse survival, but the introduction of targeted anti-HER2 therapy changed the picture: today HER2 positive patients have an improved prognosis compared to women with HER2 negative disease [27].

Here, by using an integrated database of ten previously published transcriptomic datasets, we validated the association with survival for a set of genes in non-small-cell lung cancer. Generally, the strongest associations were found for those also investigated in a previous meta-analysis (VEGF, CCNE1 and CDK1). For all of these genes higher expression was associated with shorter survival. With over 5,000 patients, the meta-analysis for VEGF [20] employed the highest number of patients – our analysis also confirmed the correlation of VEGF expression and overall survival in NSCLC patients by both univariate and multivariate analyses. The importance of VEGF is due to the availability of targeted agents directly inhibiting its activation. Interestingly, for one of the genes (CDK1) a previous meta-analysis actually rejected a correlation between the gene and survival [24]. In contrast, our results represent a large-scale independent validation of the gene. In individual genes, only a few were associated with longer survival when displaying higher expression – these include CADM1, ANXA3, ADAM28, XIAP and XAF1. Future therapeutic targeting of these will only be possible using a different approach than for most genes in which higher expression actually results in shorter survival.

After surgery, about two-thirds of recurrences for early stage disease occur at distant sites. Therefore, the eradication of micrometastases must have a high priority as early as possible. A previous meta-analysis of all the trials investigating chemotherapy benefit demonstrated a 5% improvement in overall survival [28]. This survival advantage with chemotherapy was also maintained at 9 years of follow-up. For these reasons the use of adjuvant chemotherapy is the current standard of care for patients with early stage NSCLC. In our analysis system we have integrated the use of chemotherapy to enable the validation of genes specifically related to survival in chemotherapy treated patients.

A major etiological factor for lung cancer is cigarette smoking which accounts for nearly 85% of all cases. Lung cancer development is similar to other cancer types by involving a stepwise progression to a malignant transformation driven by the collective effect of genetic changes induced by inhaled carcinogens [29]. At the same time, the number of previously never-smoker lung cancer patients is also increasing [30]. Gathering new insights into the underlying mechanism and etiological factors in these patients is necessary to better understand the disease and to develop new treatment strategies [2]. In our database we had the smoking history for 1,042 patients (of these 187 never smoker) and the meta-analysis tool also includes the option to restrict to either smoker on nonsmoker cohorts of patients. Additional filtering options include the use of gender (data is available for 1,564 patients) and staging (697 patients). Combinations of these options enable to validate biomarker candidates in sub-cohorts having a size not reached by any of the previous individual studies.

Previously, within the directors' challenge project for lung adenocarcinoma, the combined use of clinical and gene expression information performed best for predicting prognosis [17]. The multivariate analysis in the online software enables to compare clinical and molecular variables. Unfortunately, not all clinical information is published for each patient - this significantly limits the potential of any multivariate analysis including both clinical and gene expression variables.

We must also mention some issues with meta-analyses that may undermine their validity - these include biases related to patient selection, to clinical heterogeneity, to different outcome measures, to methodological and statistical techniques [31]. One option the test for biases is plotting the sample size against the effect size as this is usually skewed and asymmetrical in the presence a bias [32]. Basically, without a bias, the largest variation should be observed most in the small studies and least in large studies. This is the concept of the original funnel plot which we employed to demonstrate the correlation between hazard rates and sample sizes for two selected genes. We added an analysis option to our tool to run the computations in each dataset separately to enable swift construction of such analyses for any gene.

Finally, we have also assessed previously published gene expression signatures to predict survival. Today, the clinical application of multigene signatures is still controversial, as many of them do not outperform prognostication using conventional parameters. Here, out of seven signatures, two were capable to predict survival in stage I [13], and in all NSCLC patients [14].

In summary, by utilizing genome-wide microarray datasets published in the last five years, we have successfully integrated a large scale database suitable for the *in silico* validation of biomarker candidates in non-small cell lung cancer.

## Supporting Information

Table S1 List of genes involved in previously published gene sets.
(XLS)

R Script S1 R script used to generate Kaplan-Meier plots
(R) Click here for additional data file.
