# Correction: Online Survival Analysis Software to Assess the Prognostic Value of Biomarkers Using Transcriptomic Data in Non-Small-Cell Lung Cancer

**DOI:** 10.1371/journal.pone.0111842

**Published:** 2014-10-22

**Authors:** The PLOS ONE Staff

## Notice of Republication

This article was republished on September 17, 2014, due to the release of confidential or copyrighted information. Please download the article again to view the corrected version.
